# Variation in Milk Composition and Fatty Acid Profile during the Lactation of Araucana Creole Ewes in a Pasture-Based System

**DOI:** 10.3390/ani10010092

**Published:** 2020-01-06

**Authors:** Karla Inostroza, Silvana Bravo, Giovanni Larama, Camila Saenz, Néstor Sepúlveda

**Affiliations:** 1 Faculty of Agricultural Science and Forestry, Universidad de La Frontera, Av. Francisco Salazar 01145, Temuco P.O. Box 54-D, Chile; karla.inostroza@ufrontera.cl; 2 Institute of Animal Production, Faculty of Agricultural Sciences, Universidad Austral de Chile, Valdivia P.O. Box 567, Chile; silvana.bravo@uach.cl; 3 Centro de Modelación y Computación Científica, Universidad de La Frontera, Av. Francisco Salazar 01145, Temuco P.O. Box 54-D, Chile; giovanni.larama@ufrontera.cl; 4 Doctorado en Ciencias Agroalimentarias y Medioambiente, Universidad de La Frontera, Av. Francisco Salazar 01145, Temuco P.O. Box 54-D, Chile; csaenzmoreno@gmail.com

**Keywords:** local breed sheep, fatty acid, milk

## Abstract

**Simple Summary:**

The Araucana creole sheep is a local Chilean genetic resource and represents an important economic resource for the Mapuche ethnic group, who mainly use it for meat and wool production. In recent years, an interesting gourmet market has emerged for milk products derived from sheep’s milk. These products are highly valued because they come from sheep fed in systems based on pastures that are considered healthier than the diet of sheep fed indoors. Therefore, it is important to know the production and composition of Araucana creole ewe’s milk, which has not been studied. This information may provide an alternative for the diversification of sheep production by small farmers, contributing to the agricultural and cultural diversity of southern Chile.

**Abstract:**

Araucana creole sheep are a local animal genetic resource adapted to environmental conditions in rural production systems in southern Chile. The aim of the present study was to analyze the milk yield and composition of Araucana creole ewe’s milk from ewes maintained in a traditional grazing system of natural pastures. Twenty healthy single-bearing Araucana creole ewes were selected immediately after lambing (body condition score (BCS) of 2.8 ± 0.2, ewe weight (EW) of 62 ± 3.5 kg, and age of 3.8 ± 0.7 years). BCS, EW, and lamb weights were determined. Milk samples were obtained using the oxytocin technique at 10 days postpartum and then twice a month during the lactation stage (90 days). Protein, fat, lactose, total solids (TS), solid non-fat (SNF), urea contents, and fatty acid (FA) composition were analyzed. The Araucana ewe’s milk yield was lower than that of other dairy sheep but was higher than that of meat breeds. The milk fat had a higher content of oleic acid in the early lactation period, which decreased slowly according to the progress of lactation. The increase in oleic acid improved the milk health indexes during this period and thus provided a healthier milk product for human consumption than later in lactation. During lactation, higher conjugated linoleic acid (CLA) levels were obtained only at day 60. Our research suggests that Araucana creole sheep can provide high-quality milk during early lactation, which is rich in oleic acid and represents an alternative for the production of dairy products, improving the profitability of the productive systems of small-holder farmers in Chile.

## 1. Introduction

The conservation and sustainable use of local animal genetic resources has become a world priority, mainly because indigenous domestic animal populations are being affected by indiscriminate crossings with foreign specialized breeds. This has generated a loss of the variability of these local genotypes, which for long periods of natural selection and evolution formed a conglomerate of genes that are characterized by their adaptation to adverse edaphoclimatic conditions, resistance to diseases, and consumption of natural pastures [1,2].

In Chile, there are 38 sheep breeds, but only two are local: Araucana creole sheep and Chilota creole sheep. The Araucana creole sheep are distributed primarily in the Araucania region (38°54′ S, 72°40′ W) of southern Chile and represent an important economic resource in rural areas. Official records do not indicate the current Araucana creole sheep population. Instead, they identify the main breeds in the region, such as Suffolk Down and Texel, and the rest are classified as other breeds or crossbreeds. In 2011, it was estimated that the Araucana creole sheep population present in the Araucania region was 1000 animals, with an upward trend in the number. The first characterization report of this creole sheep was done by Sepúlveda [3], of ovine herds of the indigenous Mapuche farmers in southern Chile. Since then, given its characteristics of adaptation and low resource utilization, it has been widely used by small farmers. This zoogenetic resource is managed by small farmers of the Mapuche ethnic group and is mainly used for meat and wool production in a traditional pasture-based system [4]. There is evidence that this creole is adapted to the environmental conditions of southern Chile and has great maternal abilities, high prolificacy (lamb born/ewe lambing 157 ± 0.51%) and shorter anoestrus (155.1 ± 5.8 days) compared to other breeds [5,6]. It has an adult body weight of 57.8 ± 8.1 kg, a height to the rump of 59.8 ± 3.0 cm, and the commercial yield of the carcass is greater than 50%, with 59% of the regional composition pieces being the first category [7,8]. The Araucana creole sheep are similar in external appearance to the Hampshire Down sheep; however, they currently differ in their genetic structure. The Araucana creole sheep probably originated from Spanish and British herds. They currently maintain the external appearance of the British black face and show a close relationship to the Segureña breed, indicating their importance to the formation of creole breeds in America [4,5].

The Araucana creole sheep is currently threatened by the cross-breeding of its genetic material with external breeds, which endangers its conservation and makes it necessary to adopt measures to conserve and value this local genetic resource. Araucana creole sheep have adapted to their environment and can maximally produce at a minimal cost in a long-term and sustainable manner, which contributes to the agricultural and cultural diversity of the region.

Recently, several genetic studies have been carried out in Araucana creole sheep [6,7,8,9,10] that have enabled morphological characterization [7]. Furthermore, studies of carcasses [8], meat characteristics, and wool quality have been conducted, but there are no studies on milk. Sheep’s milk composition has been widely studied for the last decades, mainly in dairy sheep [11,12,13]. There are several endogenous and exogenous factors that affect ewe’s milk composition, including diet, season, climate, and physiological factors, e.g., individuality, breed, and lactation stage [13]. In Chile, there are few dairy sheep breeds because geographic restrictions prevent the rapid development of dairy sheep herds. One possibility is to evaluate local breeds that may have dairy capacity. During the last decade, Chile has seen an important growth in products based on sheep’s milk. The new preferences of the consumer enhance the opportunities for small dairies and local communities in the generation of sustainable products. Therefore, it is important to determine the capacity of this genotype for milk production. The aim of the present study is to analyze the milk yield and composition from Araucana creole ewes maintained in traditional grazing systems in natural pastures in southern Chile.

## 2. Materials and Methods

### 2.1. Animals and Study Site

This study was carried out in the Maquehue Experimental Farm of University of La Frontera, located in the Araucania Region, Chile (38°54′ S, 72°40′ W; 114 m above sea level). During the experimental year (2018), the annual average temperature was 11.2 °C. The annual rainfall was 1.157 mm and 42% of the precipitation occurred in July and August. Data were extracted from local meteorological station Maquehue and provided by Chilean Meteorological Directorate. From an Araucana creole flock (*n* = 86), the ewes were mated in March and April and lambings were during August and September. The reproductive parameters of this creole flock (season 2018) were: fertility 88 ± 0.6% (ewe lambing/ewe joined), prolificacy 143 ± 0.6, litter size 1.5 ± 0.4, and mortality 16.5 ± 0.3% (lamb death/lamb born). A total of 20 healthy single-bearing ewes were selected immediately after lambing, with body condition scores (BCS) of 2.8 ± 0.2, body weights of 62 ± 3.5 kg, and aged 3.8 ± 0.7 years. The lambs stayed continuously with the ewes. The Araucana creole ewes and their lambs were weighed every two weeks on a digital scale, with a 200 kg capacity and accuracy of 0.05 kg, from day 10 to 90, and BCS estimates were simultaneously made on a scale of five points, from 1 (thin) to 5 (obese) [14].

The diet of ewes was based on natural pasture and the botanical, fatty acid (FA) composition, and dry matter (DM) total availability of the pasture are presented in Table 1. The stocking rate was set to 13 sheep Ha^−1^, rotationally grazed [15]. Samples of pasture were collected every month and the herbage samples were air-oven dried at 65 °C for 48 h [16]. The natural pasture had a lower DM yield in July (309.1 kg DM ha^−1^) and a higher yield in October (1931.6 kg DM ha^−1^). The most common forage species (Table 1) in the months of July and October were perennial ryegrass (*Lolium perenne*), hairy cat’s ear (*Hypochaeris radicata*), white clover (*Trifolium repens* L.), and common sheep sorrel (*Rumex acetosella*). For the FA composition of the natural pasture, a sample of 300 mg of DM was used for lipid extraction according to the method of Burja et al. [17]. The most common FA in the pasture were C18:3n3, C18:2n6c, and C16:0.

The creole ewes were all kept in the same paddock and were under the same management conditions during the trial. This study was approved by University of La Frontera Scientific Ethical Committee (ethical review number 101_17).

### 2.2. Milk Samples

Milk yield was measured at 10 days postpartum and then twice a month during the lactation stage (90 days). The milk samples were obtained using the oxytocin technique [18,19]. The lambs stayed continuously with the ewes but were separated for the milk-recording days. The ewes were injected in the jugular vein with 2 iu oxytocin (Neurofisin, DragPharma, Santiago, Chile). Immediately after injection, they were hand milked until no more milk could be extracted from their udder. The ewes were returned to the pasture, while the lambs were kept in a pen in a separate room. After 4 h, the procedure was repeated, the second milking was recorded, and two representative milk samples (10 mL) were collected of each ewe for analysis. One of the aliquots was refrigerated at 4 °C to determine milk composition and the second aliquot was frozen at −20 °C to determine fatty acid (FA) composition. For each ewe, 24 h milk production was calculated relative to the exact time interval between milkings [20]. After milking, the ewes and lambs were returned to the pasture.

### 2.3. Milk Composition Analysis

A milk sample (10 mL) was analyzed for protein, fat, lactose, TS, SNF, and urea using Fourier infrared analysis with a MilkoScan 4000 (FOSS, Hillerod, Denmark), according to standard ISO 9622:2013 IDF 141 [21] and calibrated monthly with reference material for the different parameters analyzed, which were provided by the Laboratory for Quality Assurance of Measurement (LACM) ICYTAL of the University Austral of Chile. The samples were analyzed by the Milk Quality Laboratory (Agricultural Research Institute, Carillanca, Vilcún, Chile).

### 2.4. Milk FA Analysis

For milk fat extraction, the Röse-Gottlieb method was applied [22]. Fatty acid methyl esters (FAME) were prepared using 1.3 mL potassium hydroxide in 2 N methanol and 0.8 mL n-hexane, shaken for 30 min, and the supernatant was filtered with anhydrous sodium sulfate for analysis. FAME separation and quantification were performed using a gas chromatograph (GC). One microliter of FAME for each sample was injected with split injection mode into a Perkin Elmer GC-equipped with a flame ionization detector (Clarus 500, Perkin Elmer, Buckinghamshire, UK) and a fused silica capillary column SP^TM^ 2380 (60 m × 0.25 mm × 0.2 μm film thickness; Supelco, Pennsylvania, PA, USA). The injection and detector temperatures were 250 °C. Nitrogen was used as a carrier gas. The initial oven temperature was set at 150 °C, after 1 min the temperature was increased at a rate of 1 °C min^−1^ to 168 °C, held for 11 min, then increased at 6 °C min^−1^ to 230 °C, and then held for 8 min. FAMEs were identified by comparison with the standard FAME Mix C4-C24 (Supelco, Pennsylvania, PA, USA), which contains 37 FAMEs, analyzed under the same conditions. For the identification of conjugated linoleic acid (CLA) isomers, octadecadienoic acid and conjugated methyl ester (CLA Sigma-Aldrich, Milwaukee, WI, USA) were used as a standard. The composition of each FA was expressed as the weight percentage (percentage of total FAME).

### 2.5. Statistical Analysis

A model of the lactation curve [23] (1) for dairy cattle milk production was used to describe curves for milk production. The algebraic model was:

y_n_ = a n^b^ e^−c n^, (1)

where y_n_ is the average daily yield of the nth day and a, b, and c are constants. The average content of FAME compounds in milk samples from single-bearing ewes was calculated from GC determinations. EW, BCS, lamb weight, milk yield and composition, and FA profile for milk samples were statistically analyzed using analysis of variance (ANOVA), and days in milk as a repeated measure. The averages according to stage of lactation were compared with the Tukey test, using the general linear model (PROC GLM) of the SAS programs (Statistical Analysis System, North Carolina, NC, USA). Significant differences were considered at *p* < 0.05. Atherogenicity (AI) and thrombogenicity (TI) indices were calculated as proposed by Ulbricht and Southgate [24].

## 3. Results

### 3.1. BCS, Ewe Weight (EW), and Lamb Liveweight

Table 2 shows the variation in EW and BCS of single-bearing Araucana creole ewes during a 90 day lactation period. During this period, the Araucana creole ewes showed no significant changes in EW or BCS. Additionally, the lamb liveweight at birth was 3.98 ± 0.59 kg and increased with age (*p* < 0.05).

### 3.2. Milk Yield and Composition

**Figure 1:**
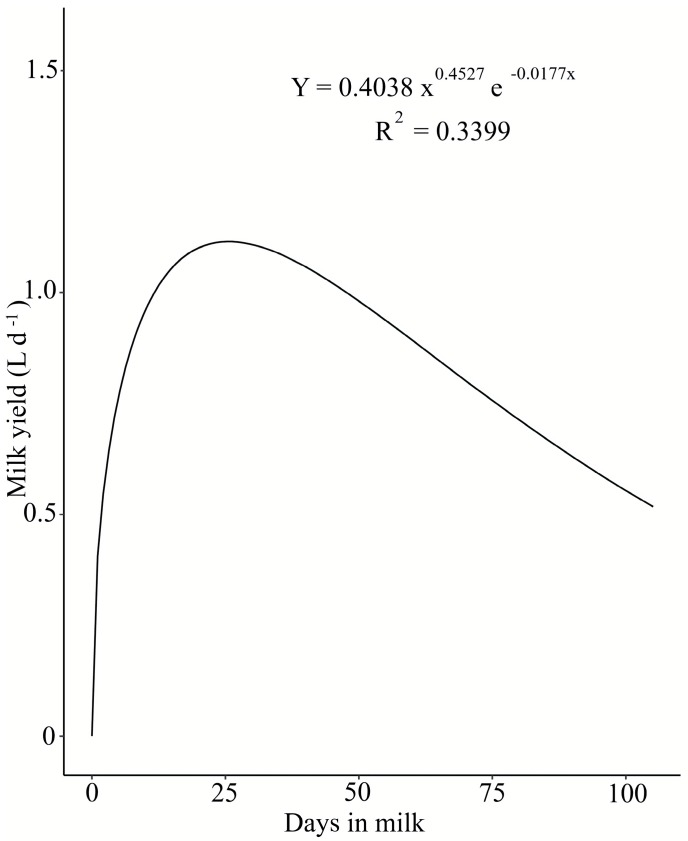
Milk yield (L day^−1^) of Araucana creole ewes (*n* = 20), during days 1–90 of lactation, predicted by Wood’s equation [23].

The single-bearing Araucana creole ewes selected for the present study produced a milk yield of 39% in the first 30 days of the lactation period (Figure 1). They reached peak milk production at day 30 (1.40 ± 0.3 L day^−1^). As expected, milk yield decreased significantly as a function of time. The Araucana creole ewes’ milk yield was estimated based on the lambs’ weight at birth (3.98 ± 0.59 kg) and at 30 days of age. For 1 kg of body weight gain, a consumption of 4.5 kg of milk was assumed [25]. The approximate milk yield in the first four weeks was 27.9 ± 2.8 L.

The milk composition (protein, fat, lactose, TS, SNF content, and urea) changed during the 90 day lactation period (Table 3). The major change in milk components occurred during the first 30 days, coinciding with the highest milk yield in Araucana creole ewes, with a decrease in fat, protein, TS, and SNF contents and an increase in lactose content, with the highest lactose content at day 60, which then decreased as the milk yield diminished. In this study, the relationships between different milk components showed that fat content was correlated with both TS and SNF content (r = 0.92; *p* < 0.01 and r = 0.14; *p* < 0.01, respectively). Additionally, the protein content was positively correlated with fat (r = 0.34; *p* < 0.01), ST (r = 0.66; *p* < 0.01), and SNF (r = 0.95; *p* < 0.01) contents.

### 3.3. FA Composition

Analysis of the milk from Araucana creole ewes indicated that over 87% of the lipid composition was present as four major FAs: myristic C14:0, palmitic C16:0, stearic C18:0, and oleic acids C18:1n9c. Capric (C10:0), palmitic, and oleic acids were the most abundant in short- (SCFAs), medium- (MCFAs), and long-chain FAs (LCFAs), respectively (Table 4). In the SCFA fraction, we observed differences in C6:0 and C10:0; C6:0 decreased and C10:0 increased at day 90. C16:0 was the most abundant MCFA in ewes, followed by C14:0, while C13:0 was detected at the lowest level. Most MCFAs tended to increase at day 90, except for C17:0 and C17:1. The sum of SCFAs and MCFAs was lower during the early lactation period and increased gradually until day 90 (*p* < 0.05). In contrast, the principal fraction in ewe’s milk, LCFA, decreased from early lactation to day 90 (*p* < 0.05). C18:1n9c, the most abundant MCFA, decreased by 9.4% on average from day 10 to 90. The concentrations of the preformed FAs, linoleic (C18:2n6c) and eicosapentaenoic acid (C20:5n3), varied between day 10 and 90.

Table 4 shows the proportions of SFAs, MUFAs, and PUFAs during lactation. Among the SFAs, myristic, palmitic, and stearic acids represented the major SFAs. The SFA content increased gradually from day 10 to day 90 (+8.6%). Among the MUFAs, the major FA was oleic acid; it represented 92–95% of MUFAs. The MUFA content decreased from 5.0% between day 10 and 30 and 8.9% between day 10 and day 90. The contents of detected PUFAs were lower than MUFAs; the total PUFA content was not affected by the lactation stage.

Different milk FAs are known to have beneficial (most unsaturated FAs, i.e., oleic acid) or adverse (some SFAs, i.e., myristic and palmitic acids) effects on health maintenance and disease prevention [13]. Ulbricht and Southgate [24] proposed two indexes, IA and IT, that allow for the comparison of different foods considering the content of atherogenic SFAs (C12:0, C14:0, and C16:0) and discarding the ratio of PUFAs to SFAs because only three FAs are hypercholesterolaemic. Fat with a higher IA value is considered more detrimental to human health. Additionally, due to the changes mentioned above, the health indexes varied with the stage of lactation, with lower values at day 10 and higher values at day 90 (Table 4).

The hypercholesterolaemic/unsaturated FA (HFA/UFA) and hypocholesterolaemic/hypercholesterolaemic (h/H) ratios also consider the hypo- and hypercholesterolaemic FAs. At day 10 of lactation, we observed a lower HFA/UFA ratio and a higher h/H ratio than at day 90 due to changes in the major FAs.

## 4. Discussion

Ewe’s milk is very important in many countries, and breeding programs are developed based on the milk yield of ewes. Ewe’s milk is high in fat and protein and is mainly used in commercial- or artisanal-quality cheeses and yogurts [26]. Traditional sheep production in Chile is based on wool and meat and is conducted by small-holder farmers. Therefore, alternatives to diversify production are indispensable. In this sense, characterizing the milk production and composition from Araucana creole ewes provides an opportunity to generate profitability within these systems through the generation of new products.

The determination of the lactation curve in creole ewes such as Araucana can be of interest. The total milk yield calculated from the lactation curve (Figure 1), according to Wood [23], averaged 81.80 and 90.39 L day^−1^ at day 90 and 105, respectively. Under similar conditions of grazing on natural pastures, the total milk yield at day 105 for other breeds, such as Chilota (genetically related to Spanish dairy sheep) and Suffolk (considered a meat breed) ewes, in south Chile averaged 113.5 and 67 L, respectively [27]. The lactation curve varies as a consequence of seasonal variation in the natural pastures. Early lactation, August and September (months in which Araucana creole sheep births are concentrated), coincided with the end of the cold rainy season and with the increasing pasture DM yield. In the middle of the lactation period, the forage yield and quality were high, and finally, the late lactation stage coincided with the end of spring and the dry season in southern Chile. The FA composition of the pastured species varied according to the vegetative stage and changed the content of healthy FAs in milk fat and thus altered the FA composition of the milk products. In the present study, the FA composition of the pasture in winter (July and August) and spring (September and October) varied mainly in MUFA and PUFA contents. The FA composition in the spring season tended to show a higher percentage of MUFA (due to the increase in oleic acid) and lower PUFA content (due to the increase in C18:2n6c and decrease in C18:3n3 FAs) than the winter season. Cabiddu et al. [28] mentioned that as the plants passed from the vegetative to the reproductive phase, linoleic acid (C18:2n6c) increased, while α-linoleic acid (C18:3n3, ALA) decreased. The pasture plays a key role in improving the health benefits of ewe’s milk due to the marked effects of pasture on the milk fat ALA and CLA contents [28,29]. Thus, the results should be considered according to the conditions in southern Chile presented in this research and the annual/seasonal variation in pasture during the research season.

Farmers greatly value the early maturation of Araucana creole for meat production, and one important characteristic of this creole sheep is its outstanding food conversion ability, which enables it to survive well in marginal grass conditions [5]. The daily live weight gain of creole lambs between 30–60 days and 60–90 days was 0.19 kg day^−1^ and 0.17 kg day^−1^, respectively. As mentioned above, these farming systems depend on pasture yield, which was high in quantity and quality in the period between 30 and 60 days but began to decrease between 60 and 90 days, which agrees with the decrease in lamb daily liveweight gain in this period.

This study is the first report of Araucana creole ewe’s milk yield and composition. The lactose percentage reached its peak at day 60 and related to the lowest fat and TS contents. The protein content decreased before the other components at day 30, coinciding with the maximum milk yield. Bencini and Purvis [30] observed that fat, protein, and TS concentrations were high at the beginning of lactation, while the concentration of lactose followed the curve of milk production in Merino sheep. Kay et al. [31] reported similar variations from weeks 1 to 4 in Holstein cows. The values of milk composition are coincident with those reported by Park et al. [32] in sheep’s milk. A comparison between different breeds in the central-south zone of Chile indicated that Suffolk and local sheep present a similar variation in milk composition during lactation. The milk composition during the lactation period of Araucana creole ewes (fat: 6.6%, lactose: 5.3%, and TS: 18.2%) is similar to other local sheep (fat: 6.6%, lactose: 5.3%, and TS: 18.5%); however, the protein content is lower in Araucana creole milk (protein: 4.6%) compared to the local sheep (protein: 6.1%) and Suffolk sheep (protein: 6.0%) [33]. In this study, the relationships between different milk components showed that fat was correlated with both TS and SNF content (r = 0.92; *p* < 0.01 and r = 0.14; *p* < 0.01, respectively). Additionally, protein content was positively correlated with fat (r = 0.34; *p* < 0.01), TS (r = 0.66; *p* < 0.01), and SNF content (r = 0.95; *p* < 0.01).

Overall, we observed large changes in the FA composition of Araucana creole ewe’s milk during lactation. According to Garnsworthy et al. [34], the FA profile varies with the stage of lactation, which influences the yield of the FAs. The stage of lactation might also influence the relative proportions of individual FAs indirectly by influencing the balance between body fat mobilization and de novo synthesis of FAs in the mammary glands. In early lactation, when the energy balance is negative [29,35], milk fat concentration and the concentration of preformed long-chain FAs increases due to the uptake of non-esterified FAs derived from body fat mobilization [36]. During this period, lactating Araucana creole ewes may have restricted nutrient intake, as the pastures begin to increase in DM, resulting in the need for supplementation with grains, and this restricted nutrient intake may result in body weight loss and low BCS. In the early stage of lactation, the SCFA fraction and lauric (C12:0), tridecanoic (C13:0), and myristic acids corresponded to the proportion of de novo synthesis in the mammary glands. This is consistent with another study by Palmquist et al. [37], which reported lower proportions of these FAs in early lactation because de novo synthesis of these FAs was inhibited by LCFA from body fat. In the present study, LCFA levels were higher than SCFA and MCFA levels during the early lactation period (*p* < 0.05). In early lactation, we observed a lower content of stearic acid and a higher content of oleic acid. Bitman and Wood [38] noted a consistent compensatory decrease in the oleic acid concentration in mature milk, and it was the major FA that compensated for the increased quantities of C10:0, C12:0, C13:0, C14:0, and C16:0. Thus, when the LCFA proportion decreased in mature milk at day 90, the proportions of SCFAs and MCFAs increased (*p* < 0.05). Levels of metabolically valuable SCFAs and MCFAs (C6:0, C8:0, C10:0, and C12:0) are significantly higher in sheep and goat milk compared to cow milk. These FAs are associated with the characteristic flavours of cheeses and can also be used to detect admixtures of milk from different species [32].

Among the LCFAs, the major FA group in milk, oleic acid, decreased from early lactation to day 90 (*p* < 0.05). According to Rukkwamsuk et al. [39], oleic acid, the predominant FA in adipocytes, is the primary FA released during lipolysis. Oleic acid represents approximately 25% of the total fat in cow’s milk, and dairy products are the main source of FAs in the human diet in many countries, which contributes to decreases in plasma cholesterol and triglycerides [40]. Research in managing pastures and the FA composition in sheep that were fed forages [28,41] indicated that oleic acid is affected by the forage species and seasonal stage, varying between 13.28% and 25.36% in winter and 14.81% and 41.30% in spring. These differences were transferred to the FA composition of 60-day-old cheeses obtained from sheep’s milk produced during spring, which contains a higher oleic acid content compared to milk produced in the winter (40.99 g 100 g^−1^ and 23.83 g 100 g^−1^, respectively). In this study, the highest oleic acid concentration in milk fat was obtained in late winter and early spring, and it decreased as lactation progressed. The oleic acid decreased by 25% from week 1 to 8 in cow milk [39]. Moreover, in Araucana creole ewe’s milk, oleic acid only decreased 9.4% from day 10 to 90; therefore, there is a high content of this acid throughout lactation. Moreover, some oleic acid is produced from stearic acid in the mammary glands by the Δ9-desaturase enzyme system [34].

Sheep’s milk fat is mainly composed of saturated FAs (SFAs; 66%), followed by monounsaturated (MUFAs) and polyunsaturated FAs (PUFA) (28% and 6%, respectively) [42]. Araucana creole ewe’s milk fat contained on average 58.9% SFAs, 36.5% MUFAs and 4.8% PUFAs after 90 days of lactation. The SFAs are principally represented by SCFAs and MCFA, the main fraction in Araucana creole ewe’s milk during lactation and increasing until day 90, largely caused by the increase in MCFA content. The decreased MUFA proportion during lactation is due to the decrease in oleic acid, as mentioned above.

Much attention has been directed towards CLA because of its anticarcinogenic properties. In milk fat, cis-9, trans-11 CLA composes 75–90% of total CLA, whereas trans-10, cis-12 CLA constitutes a minor isomer [43]. In Araucana creole ewe’s milk fat, the CLA content was higher at day 60 compared to the other lactation stages. The effect of pastures on the milk fat ALA and CLA content is related to the high ALA content in green pastures, which is partially biohydrogenated in the rumen and then secreted into milk, as well as partially converted into CLA in the mammary tissue by stearoyl CoA desaturase [29]. In this study, there were no differences in ALA content (*p* > 0.05), while the CLA content varied throughout the lactation period. A high CLA content in sheep milk was obtained when the animals were fed on mixed pastures (18.85–20.44 mg g^−1^ of fat) [28]. In this study, the higher CLA milk fat content at day 60 was accompanied by decreased SFAs (C12:0, C14:0, and C16:0) and oleic acid content in milk fat. Kay et al. [31] reported that cis-9, trans-11 CLA concentrations increased from week 1 to 16 in Holstein cows. In contrast, Kelsey et al. [44] reported that milk fat CLA content was not affected by the stage of lactation, but that tissue desaturase activity, isomerization, and biohydrogenation may be the contributing factors to CLA content. Comparatively, production variables, including stage of lactation, milk fat content, and milk fat yield, have little to no effect on the CLA content of cow milk [43]. According to Soják et al. [13], the average CLA content of grazing ewes was 15.4 mg g^−1^, which is lower than that of Araucana ewe’s milk fat CLA (17.2 mg g^−1^).

Additionally, the IA, TI, HFA/UFA, h/H PUFA/SFA, and n-6/n-3 ratios were estimated. These indexes are used to value the nutritional and health aspects of animal fat for consumers. In this study, we found higher AI and TI indexes at day 90 compared to day 10. These differences were related to the increased atherogenic SFAs at day 90 of lactation and lower levels of C18:1n9c in milk fat. Additionally, the HFA/UFA and h/H ratios, which consider the atherogenic SFAs and the hypocholesterolaemic effect of C18:1n9c and PUFAs, respectively, were within considerably healthy ranges at day 10. The observation that Araucana creole ewe’s milk fat in the late lactation stage had higher levels of SFAs and higher AI and TI and lower MUFA content implies that milk at this stage may be less healthy for human consumption and for the production of dairy products. A PUFA-to-SFA ratio above 0.45 and a ratio of n-6/n-3 PUFA below 4.0 are recommended [45]. Our results revealed a PUFA/SFA ratio (0.07–0.09) considerably lower than 0.45 and a ratio of n-6/n-3 PUFA (1.49–1.92) within the recommended levels. There is a debate about the optimal dietary ratio of the parent n-6 FA linoleic acid to the n-3 FA ALA to promote efficient conversion of ALA to eicosapentaenoic and docosahexaenoic acid, both of which have positive implications for human health [46]. In the present study, linoleic acid decreased towards the end of lactation, while ALA remained constant; thus, the lowest ratio of these FAs was observed when milk production decreased. In ewes grazing part-time, the values of the PUFA/SFA and MUFA/SFA ratios in milk are significantly higher compared with the milk of ewes fed indoors [47].

Initially, Ulbricht and Southgate [24] indicated some SFA to be more detrimental, and the effects that FAs might have on human health. SFA acts to increase LDL-cholesterol and elevate cardiovascular disease (CVD) risk. However, the pathogenic role of SFA has become highly controversial in recent years, as the intake of SFA also increases high-density lipoprotein cholesterol (HDL), offsetting the adverse effects of elevated low-density lipoprotein cholesterol (LDL) [48]. Additionally, recent scientific studies have not linked dairy products consumption with an increased CVD in a healthy population [49]. Thus, these indexes can be used as a reference and recommendation for consumers with established CVD risk.

## 5. Conclusions

Based on the results, we conclude that for Araucana creole sheep-farming systems where diet is based principally on pasture grazing, the milk fat can have important amounts of healthy FAs, which vary with seasonal changes in the pasture composition. During early lactation, we observed a higher content of MUFAs and a lower SFA content than during other lactation periods. The contribution to the MUFA content is largely attributed to oleic acid, which is higher in the early lactation period and, therefore, an increase in this beneficial FA could improve the health indexes. These data indicate that Araucana creole ewe’s milk could be healthier during this period for human consumption and the production of dairy products. Araucana creole ewe’s milk represents an alternative to diversify pasture-based production systems with the development of dairy products that may be perceived to be healthier for a growing gourmet market in southern Chile.

## Figures and Tables

**Table 1:** Pasture botanical and fatty acid composition in Araucana creole sheep grazing systems in the valley of Araucania Region.

Species	July	August	September	October
*Lolium perenne* (%)	49.5	43.0	42.1	40.6
*Hypochaeris radicata* (%)	2.4	8.9	10.2	15.8
*Trifolium repens* (%)	15.4	9.9	8.5	7.9
*Rumex acetosella* (%)	1.2	5.3	6.9	7.7
*Others* (%)	31.5	32.9	32.3	28
Saturated fatty acids (%)	24.0	25.3	25.8	28.3
Monounsaturated fatty acids (%)	3.5	4.8	8.3	10.1
Polyunsaturated fatty acids (%)	72.5	69.9	65.9	61.6
Dry matter (kg ha^−1^)	309.1	600.0	1450.0	1931.6

**Table 2:** Ewe weight (EW) and body condition score (BCS) of Araucana creole ewes (*n* = 20) during lactation and liveweight of Araucana creole lambs (*n* = 20).

Variable	Days in Milk				SEM	*p*-Value
	10	30	60	90		
EW	53.88	50.54	53.17	54.58	1.14	0.28
BCS	2.18	2.21	2.50	2.58	0.05	0.08
Lamb weight	5.97 ^d^	10.18 ^c^	16.02 ^b^	21.00 ^a^	0.83	0.00

SEM: standard error of the mean; ^a,b,c,d^ means with different superscript are significantly different (*p* < 0.05).

**Table 3:** Yield and composition of Araucana creole ewe’s milk (*n* = 20) during lactation.

Variable	Days in Milk				SEM	*p*-Value
	10	30	60	90		
Production (L day^−1^)	0.95 ^b^	1.40 ^a^	0.74 ^b^	0.68 ^b^	0.07	0.02
Protein (%)	4.09 ^c^	4.30 ^c^	4.77 ^b^	5.24 ^a^	0.11	0.001
Fat (%)	7.09 ^a^	6.31 ^a,b^	5.76 ^b^	7.07 ^a^	0.21	0.001
Lactose (%)	5.28 ^a,b^	5.36 ^a,b^	5.46 ^a^	5.16 ^b^	0.04	0.001
Total solids (%)	18.19 ^a,b^	17.68 ^b^	17.59 ^b^	19.28 ^a^	0.26	0.001
Solid non-fat (%)	10.48 ^b^	10.80 ^b^	11.30 ^a^	11.58 ^a^	0.10	0.001
Urea (g 100 mL^−1^)	0.04 ^b^	0.05 ^a^	0.04 ^b^	0.05 ^a^	0.001	0.001

SEM: standard error of the mean; ^a,b,c^ means with different superscript are significantly different (*p* < 0.05).

**Table 4:** Mean content (percentage of total FAME) of individual fatty acids (FAs) in the milk fat of Araucana creole ewes (n = 20) during 90 days of lactation.

Fatty Acid	Days in Milk				SEM	*p*-Value
	10	30	60	90		
C6:0	0.17 ^a^	0.09 ^b^	0.09 ^b^	0.09 ^b^	0.01	0.01
C8:0	0.28	0.19	0.24	0.25	0.08	ns
C10:0	1.65 ^c^	1.99 ^b,c^	2.87 ^a,b^	3.24 ^a^	0.09	0.01
C12:0	1.35 ^c^	1.71 ^b,c^	2.19 ^ab^	2.57 ^a^	0.06	0.02
C13:0	0.06 ^c^	0.08 ^c^	0.13 ^b^	0.17 ^a^	0.005	0.01
C14:0	5.20 ^c^	6.26 ^b,c^	6.87 ^b^	9.30 ^a^	0.17	0.01
C14:1	0.55 ^d^	0.83 ^c^	1.25 ^b^	1.57 ^a^	0.04	0.01
C15:0	0.15	0.20	0.18	0.15	0.01	ns
C16:0	24.14 ^b^	24.80 ^b^	24.30 ^b^	26.75 ^a^	0.16	0.02
C16:1	1.10 ^b^	1.02 ^b^	1.30 ^a^	1.07 ^b^	0.03	0.04
C17:0	1.17 ^a^	1.01 ^ab^	0.95 ^b^	0.98 ^b^	0.02	0.01
C17:1	0.27 ^a^	0.15 ^b,c^	0.11 ^c^	0.18 ^b^	0.01	0.01
C18:0	20.50 ^b,c^	23.11 ^b^	24.86 ^a^	18.35 ^c^	0.38	0.03
C18:1n9t	0.16 ^b^	0.31 ^a^	0.34 ^a^	0.17 ^b^	0.01	0.02
C18:1n9c	38.74 ^a^	33.19 ^b^	29.90 ^c^	29.39 ^c^	0.48	0.01
C18:2n6c	1.96 ^a^	1.54 ^b^	1.21 ^c^	1.47 ^b^	0.02	0.01
C18:3n6	0.15 ^d^	0.22 ^c^	0.27 ^b^	0.43 ^a^	0.01	0.01
C18:3n3	0.94	0.92	0.83	0.89	0.01	ns
Total CLA ^1^	1.28 ^b^	1.80 ^ab^	1.91 ^a^	1.71 ^a,b^	0.04	0.04
C21:0	0.04 ^c^	0.10 ^a^	0.06 ^b^	0.06 ^b^	0.003	0.01
C20:5 n3	0.13 ^a^	0.09 ^b^	0.13 ^a^	0.14 ^a^	0.01	0.02
C24:1n9	0.02 ^c^	0.02 ^c^	0.04 ^b^	0.09 ^a^	0.002	0.02
C22:6 n3	0.03 ^b^	0.03 ^b^	0.03 ^b^	0.05 ^a^	0.001	0.03
SCFA	2.10 ^c^	2.27 ^c^	3.20 ^b^	3.58 ^a^	0.02	0.01
MCFA	33.97 ^c^	36.06 ^b^	37.28 ^b^	42.74 ^a^	0.05	0.01
LCFA	63.95 ^a^	61.33 ^a^	59.58 ^a^	52.75 ^b^	0.06	0.01
SFA	54.70 ^c^	59.55 ^b^	62.69 ^a,b^	63.34 ^a^	0.53	0.00
MUFA	40.83 ^a^	35.82 ^b^	32.94 ^c^	31.91 ^c^	0.52	0.00
PUFA	4.48	4.63	4.37	4.75	0.68	ns
IA	1.02 ^c^	1.27 ^b,c^	1.47 ^a,b^	1.73 ^a^	0.04	0.01
IT	1.12 ^b^	1.40 ^ab^	1.58 ^a^	1.64 ^a^	0.03	0.01
PUFA/SFA	0.08 ^a^	0.08 ^a^	0.07 ^b^	0.07 ^b^	0.001	0.001
MUFA/SFA	0.75 ^a^	0.60 ^b^	0.53 ^c^	0.50 ^c^	0.01	0.001
n-6/n-3 PUFA	1.92 ^a^	1.69 ^b^	1.49 ^c^	1.76 ^a,b^	0.03	0.02
HFA/UFA	0.68 ^c^	0.81 ^b^	0.89 ^a^	1.05 ^a^	0.02	0.00
h/H	1.47 ^a^	1.22 ^b^	1.09 ^c^	0.95 ^c^	0.03	0.03

^1^ Total CLA: conjugated linoleic acid, isomers cis-9 trans-11/trans-9 cis-11; SEM: standard error of the means; SCFA: short-chain FAs (sum of C6:0 to C10:0); MCFA: medium-chain FAs (sum of C12:0 to C17:1); LCFA: long-chain FAs (sum of C18:0 to C22:6n3); SFA: saturated FAs; MUFA: monounsaturated FAs; PUFA: polyunsaturated FAs; HFA/UFA: hypercholesterolaemic (C12:0 + C14:0 + C16:0)/unsaturated fatty acids; h/H: hypocholesterolaemic/hypercholestrolaemic ratio = (C18:1 + PUFA)/(C14:0 + C16:0); NS: non-significant difference; ^a,b,c,d^ means with different superscript are significantly different (*p* < 0.05).
